# Assessment of Online Food Ordering and Delivery in Singapore During the COVID-19 Pandemic

**DOI:** 10.1001/jamanetworkopen.2021.26466

**Published:** 2021-09-23

**Authors:** Sumit Agarwal, Peiying Huang, Chenxi Luo, Yu Qin, Changwei Zhan

**Affiliations:** 1 Department of Finance, National University of Singapore, Singapore; 2 College of Management, Shenzhen University, Shenzhen, China; 3 Institute of Real Estate and Urban Studies, National University of Singapore, Singapore; 4 Department of Real Estate, National University of Singapore, Singapore

## Abstract

This cohort study examines patterns of eating habits of people in Singapore during the COVID-19 pandemic.

## Introduction

In 2020, the COVID-19 outbreak was declared to be a pandemic. Governments implemented various measures, such as social distancing, quarantine, and lockdowns, to minimize the spread and effects of COVID-19. Previous studies^1,2^ based on questionnaires showed that respondents reported unhealthier eating habits during lockdown. Unhealthy eating behavior formed during the pandemic could result in undesirable long-term health consequences, such as coronary heart disease,^3,4^ chronic diseases,^5^ and Alzheimer disease.^6^

## Methods

This cohort study was deemed exempt from full review by the faculty ethics review committee at the National University of Singapore, and informed consent was waived because of the deidentified and encrypted nature of the data. We followed the Strengthening the Reporting of Observational Studies in Epidemiology (STROBE) reporting guideline.

In this cohort study, we used an order-level data set from a Singapore-based online food ordering and delivery platform to investigate the association between Singapore’s lockdown (ie, Circuit Breaker [CB]) and eating habits. Each observation with food items was an order placed by an encrypted customer. We coded Chinese New Year (CNY) as day 1. Day −89 to day 220 of 2020 CNY was the treatment period, while day −89 to day 220 of 2019 CNY was the control period.

We estimated the difference-in-differences (DID) model, which assumed similar dietary trends in the 2 periods, to test whether customer diet changed during CB and post-CB period. In the first equation, diet*_rct_* denoted the outcome (ie, vegetable, barbecue or fried food, beverage, or dessert) of restaurant *r* and customer *c* on date *t*. Each of the outcome variables equaled 1 if any item could be matched with keywords specified in our dictionaries (eTable in the Supplement), otherwise, 0. Dummy treat*_t_* denoted 1 for the treatment period or 0 for the control period. Dummies cbperiod*_t_* and after*_t_* represented the CB period (day 74 to day 129) and the post-CB period (day 130 to day 220), respectively. Coefficients β_1_ and β_2_ were dietary changes during the 2 periods. Day −89 to day 73 was the benchmark period.


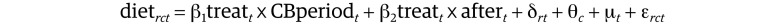


In the second equation, the sample was further divided into 31 ten-day periods (represented by *j*) and interacted with treat*_t_* to analyze the dynamic dietary changes.
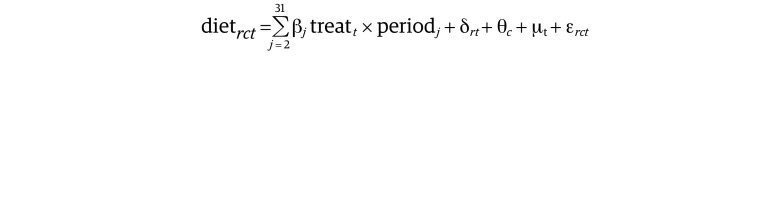
The first 10-day period was the benchmark. eFigure in the Supplement further indicated research design and model details. The software was Stata 15 (StataCorp, LLC). We performed 2-sided *t* tests, and statistical significance was set at *P* < .001. The statistical analysis was performed from August 2020 to July 2021.

## Results

This cohort study included 42 495 obversions involving 11 372 customers and 462 restaurants. Relative to the benchmark, during CB, the probability that the order contained vegetables decreased by 15% (95% CI, 12%-19%), while the probability of an order in the barbecue/fried food or beverage category increased by 11% (95% CI, 8%-14%) and 4% (95% CI, 2%-6%), respectively (*P* < .001) (Table). During the post-CB period, these patterns persisted. Dynamic changes in the Figure also showed that the probability of ordering vegetables decreased by 21% (95% CI, 6%-37%) during day 61 to day 70 when the advance work-from-home order was implemented and the lockdown was announced (*P* < .001), relative to the benchmark, and this trend persisted till the end of the sample period.

**Table.** Lockdown and Dietary Changes^a^

Sample	Vegetable		Barbecue/fry		Beverage		Dessert	
	Estimate (95% CI)	*P* value	Estimate (95% CI)	*P* value	Estimate (95% CI)	*P* value	Estimate (95% CI)	*P* value
Full sample, 42 495^b^								
Intercept	0.76 (0.75 to 0.78)	<.001	0.18 (0.15 to 0.20)	<.001	0.05 (0.04 to 0.06)	<.001	0.06 (0.05 to 0.07)	<.001
Dietary change during CB (treat*_t_* × CBperiod*_t_*)	−0.15 (−0.19 to −0.12)	<.001	0.11 (0.08 to 0.14)	<.001	0.04 (0.02 to 0.06)	<.001	0.002 (−0.02 to 0.02)	.86
Dietary change after CB (treat*_t_* × after*_t_*)	−0.21 (−0.26 to −0.16)	<.001	0.16 (0.12 to 0.21)	<.001	0.06 (0.04 to 0.08)	<.001	0.01 (−0.008 to 0.04)	.21
Sample without new customers, 25 688^c^								
Intercept	0.76 (0.75 to 0.77)	<.001	0.18 (0.16 to 0.19)	<.001	0.05 (0.05 to 0.06)	<.001	0.06 (0.05 to 0.06)	<.001
Dietary change during CB (treat*_t_* × CBperiod*_t_*)	−0.15 (−0.19 to −0.11)	<.001	0.11 (0.07 to 0.14)	<.001	0.04 (0.02 to 0.05)	<.001	−0.003 (−0.03 to 0.02)	.79
Dietary change after CB (treat*_t_* × after*_t_*)	−0.19 (−0.25 to −0.14)	<.001	0.15 (0.10 to 0.21)	<.001	0.05 (0.02 to 0.07)	<.001	0.007 (−0.02 to 0.03)	.60
Test before lockdown changes, 41 512^d^								
Intercept	0.77 (0.75 to 0.78)	<.001	0.17 (0.15 to 0.19)	<.001	0.05 (0.04 to 0.06)	<.001	0.06 (0.05 to 0.06)	<.001
Dietary change before CB (treat*_t_* × before*_t_*)	0.04 (−0.03 to 0.11)	.28	−0.05 (−0.11 to 0.02)	.15	−0.01 (−0.05 to 0.03)	.55	−0.02 (−0.06 to 0.03)	.48
Dietary change during CB (treat*_t_* × CBperiod*_t_*)	−0.11 (−0.19 to −0.03)	.01	0.06 (−0.01 to 0.13)	.08	0.03 (−0.01 to 0.07)	.17	−0.01 (−0.06 to 0.03)	.56
Dietary change after CB (treat*_t_* × after*_t_*)	−0.17 (−0.25 to −0.08)	<.001	0.12 (0.03 to 0.20)	.01	0.04 (0.001 to 0.09)	.05	−0.001 (−0.05 to 0.04)	.94

Abbreviation: CB, Circuit Breaker.

^a^ Fixed effects of customer, restaurant promotion, lunar date, day of week, and public holiday were controlled for restaurant-, customer-, and date-specific factors. Robust standard errors were clustered at customer and restaurant levels.

^b^ Baseline results based on the full sample.

^c^ Results based on the sample without new customers, which were defined as customers who placed their first orders during the treatment period.

^d^ Results after adding a pre-CB period with day 1 to day 15 were dropped to eliminate the noise in data during the Chinese New Year period.

**Figure.**
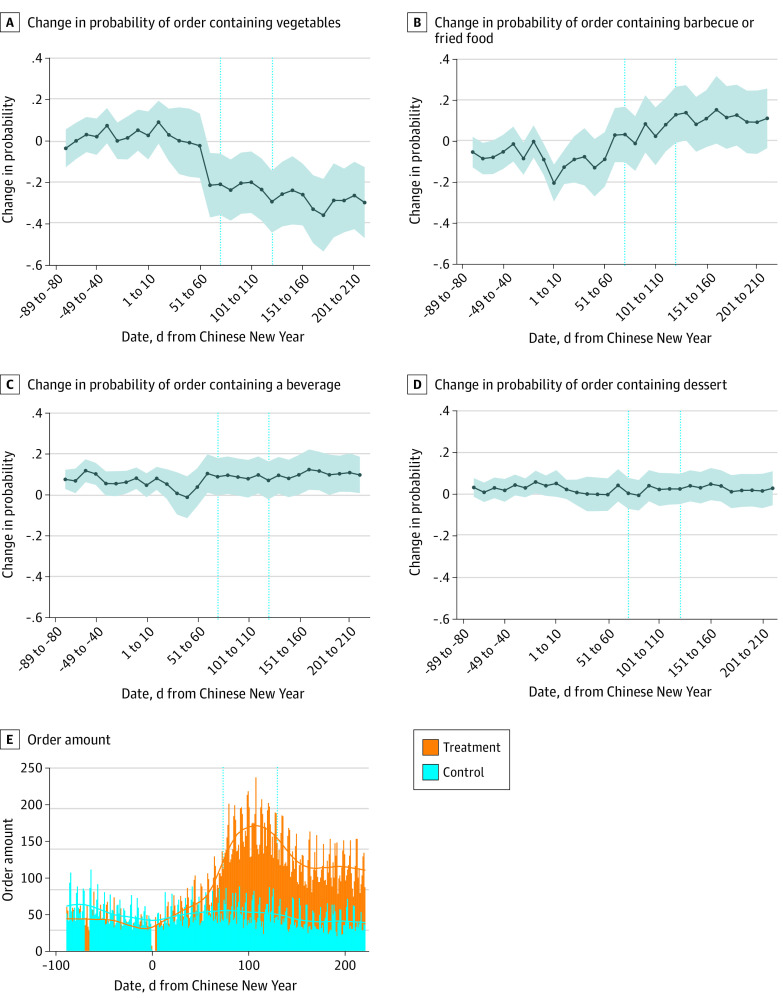
Change in Probability of Ordering Different Kinds of Food During the COVID-19 Pandemic The shaded region in panels A-D represents the 95% CI. In panel E, the bars represent the order amount (ie, how many orders were placed by customers each day). The lines are local polynomial smooth lines of order amount. The left and right vertical lines represent the beginning and end of the Circuit Breaker period.

After excluding customers who placed their first orders during the treatment period to eliminate the concerns about new customers’ distinct tastes, our results were consistent with baseline results. No significant pre-CB changes were found when we included a dummy before*_t_*, which indicated the pre-CB period between the confirmation of the first infection to the day before CB began.

## Discussion

We used a data set of real food orders instead of surveys, and this ordering data could reflect the actual changes in eating habits. Our results suggested an association between customers’ diets and unhealthier eating habits during the COVID-19 lockdown, and changes in eating habits persisted after lockdown measures were removed.

This study has limitations. We could not measure exact proportions of nutritional ingredients. More data is needed to study the long-term dietary changes and health outcomes. Our findings suggest that dietary changes occurred during and after the pandemic. Governments should properly guide the public regarding their dietary choices when implementing lockdown policies.

## Notes

Supplement. **eFigure.** Empirical Strategy **eTable.** Dictionaries of Outcome Variables
