# Supplementary material for: Assessment of Online Food Ordering and Delivery in Singapore During the COVID-19 Pandemic
Source: JAMA Netw Open. 2021 Sep 23;4(9):e2126466. doi: 10.1001/jamanetworkopen.2021.26466 (PMC8461503; doi:10.1001/jamanetworkopen.2021.26466)
Supplement: Supplement. — eFigure. Empirical Strategy eTable. Dictionaries of Outcome Variables [file jamanetwopen-e2126466-s001.pdf]

Supplemental Online Content

Agarwal S, Huang P, Luo C, Qin Y, Zhan C. Assessment of online food ordering and delivery during the COVID-19 pandemic in Singapore. *JAMA Netw Open*. 2021;4(9):e2126466. doi:10.1001/jamanetworkopen.2021.26466

eFigure. Empirical Strategy

eTable. Dictionaries of Outcome Variables

This supplemental material has been provided by the authors to give readers additional information about their work.

© 2021 Agarwal S et al. *JAMA Network Open.*

eFigure 1. Empirical Strategy

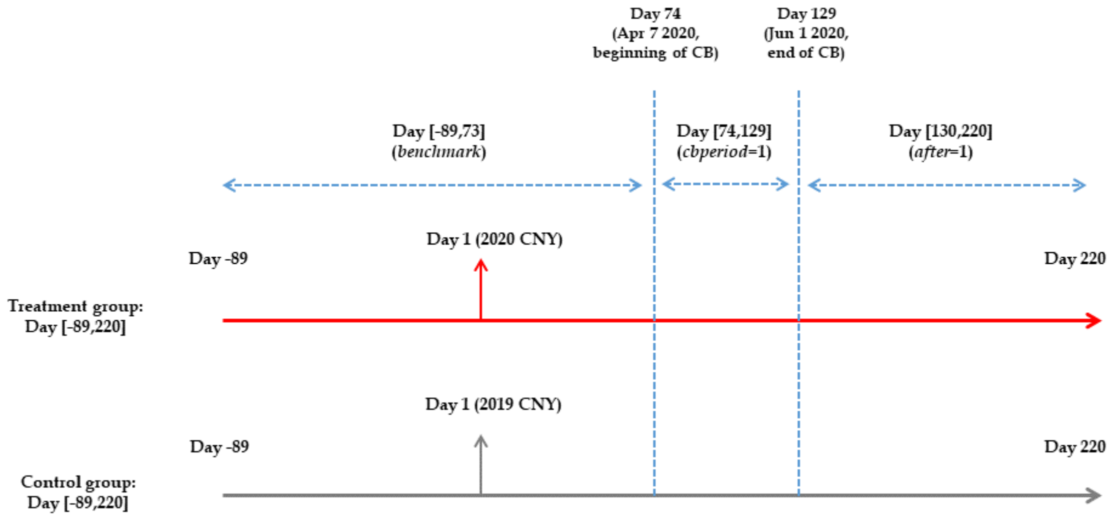

We coded order's date based on the number of days of this day to Chinese New Year (Day 1). We divided our main sample into two 310-day periods, i.e., the treatment group with orders placed between Day -89 (Oct 27, 2019) and Day 220 (Aug 31, 2020) around the 2020 CNY, and the control group, i.e., orders placed between Day -89 (Nov 7, 2018) and Day 220 (Sep 12, 2019) around the 2019 CNY. We divided the treatment group and the control group into three periods by two critical dates: Day 74 (corresponding to Apr 7 in 2020 when CB began) and Day 129 (corresponding to Jun 1 in 2020 when CB ended). Then we generated two dummy variables, cbperiod and after, to represent the CB period (Day [74, 129]) and the post-CB period (Day [130, 220]), respectively, while Day [-89, 73] was set as the benchmark period.

We used equation (1) in the main text to estimate the association between lockdown and dietary changes. We also included fixed effects in our main setting to control for restaurant-, customer-, and date-specific factors. The food delivery platform gave restaurants the authority to have their own promotions besides the platform-level uniform promotions since March 2019. Therefore, we constructed a dummy variable promotion which denotes 1 if the order was placed after Feb 2019 and otherwise 0. Then we included restaurant-promotion fixed effects δ_{rt} by interacting promotion with restaurant ID dummies. δ_{rt} captured restaurant-level characteristics, such as cooking style, location, popularity, and restaurant-specific promotion activities. θ_c indicated customer ID fixed effects, which capture customers' time-invariant characteristics, such as gender, age, tastes, consumption habits, etc. μ_t indicated a set of time-related fixed effects, including fixed effects of lunar date, day of week, and public holidays in Singapore. Lunar date was the date ID ranging from Day -89 to Day 220. The public holidays were confirmed by the Ministry of Manpower, Singapore, based on the Gregorian calendar. ε_{rct} was the error term.

© 2021 Agarwal S et al. *JAMA Network Open.*

eTable. Dictionaries of Outcome Variables

Variable	Keywords for identification	Translated keywords in English
vegetable	百合, 荸荠, 菜, 草, 椿, 豆, 甘蓝, 菇, 瓜, 海带, 茴香, 蓟, 茭白, 椒, 芥, 韭, 桔梗, 菊, 蕨, 菌, 莲, 菱, 萝卜, 苗, 蘑, 木耳, 苜蓿, 牛蒡, 藕, 茄, 芹, 秋葵, 三鲜, 山药, 上海青, 薯, 笋, 苔, 薹, 茼蒿, 莴, 芜, 西红柿, 西葫芦, 西兰花, 苋, 雪里红, 雪里蕻, 芽, 洋葱, 叶, 银耳, 油麦, 玉米, 芋, 竹	lily, water chestnut (*biqi*), vegetable (*cai*), vegetable (*cao*), Chinese toon, bean, cabbage, mushroom (*gu*), melon, kelp, fennel, thistle, cane shoot, pepper, mustard, leek, platycodon, chrysanthemum, fern, mushroom (*jun*), lotus, water chestnut (*ling*), radish, vegetable (*miao*), mushroom (*mo*), black fungus, alfalfa, burdock, lotus root, eggplant, celery, okra, *sanxian*, yam, Chinese cabbage (*shanghaiqing*), potato, bamboo shoot, garlic sprout (*tai* 1), garlic sprout (*tai* 2), garland chrysanthemum, lettuce, vegetable (*wu*), tomato, zucchini, broccoli, amaranth, potherb mustard (*xuelihong* 1), potherb mustard (*xuelihong* 2), sprout, onion, vegetable (*ye*), tremella, romaine lettuce, corn, taro, bamboo
barbecue/fry	烤, 炸	barbecue (*kao*), fry (*zha*)
beverage	juice, 北冰洋, 冰糖雪梨, 茶 π, 橙,芬达, 蜂蜜柚子, 甘蔗, 果醋, 果粒橙, 果奶, 果汁, 红牛, 加多宝, 卡布奇诺, 可乐, 酷儿, 凉茶, 六个核桃, 马蹄水, 脉动, 美禄, 美年达, 摩卡, 拿铁, 奶茶, 柠檬茶, 葡萄汁, 七喜, 雀巢, 水晶葡萄, 水蜜桃汁, 水溶 C, 酸柑, 酸梅, 哇哈哈, 王老吉, 旺仔牛奶, 雪碧, 椰树, 椰汁, 椰子汁, 薏米, 饮料, 营养快线, 竹蔗	juice, *Arctic Ocean*, crystal sugra snow pear, *Tea π*, orange, *Fanta*, honey grapefruit, sugar cane juice (*ganzhe*), fruit vinegar, *Pulpy Orange*, fruit milk, juice (*guozhi*), *Red Bull*, herbal tea (*Jiaduobao*), cappuccino, cola, *Qoo*, herbal tea (*liangcha*), walnut milk (*Liugehetao*), water chestnut juice, *Mizone*, *Milo*, *Mirinda*, mocha, latte, milk tea, lemon tea, grape juice, *7-Up*, *Nestle*, crystal grape juice, peach juice, *Water Soluble C*, lime juice, plum juice, *Wahaha*, *Sprite*, coconut milk (*yeshu*), coconut milk (*yezhi*), coconut milk (*yezizhi*), barley water, beverage, *Nutri-express Yogurt*, sugar cane juice (*zhuzhe*)
dessert	班戟, 冰粉, 冰激淋, 冰激凌, 冰淇淋, 冰淇凌, 冰沙, 饼干, 布丁, 布朗尼, 蛋糕, 蛋挞, 豆花, 豆沙, 龟苓膏, 核桃夹心, 红枣银耳, 花生糊, 焦糖, 银耳莲子, 玛芬, 慕斯, 奶酪, 奶昔, 奶油, 藕粉, 巧克力, 曲奇, 双皮奶, 松饼, 松糕, 汤圆, 糖葫芦, 糖水, 桃胶银耳, 提拉米苏, 土司, 西米捞, 西米露, 仙草, 鲜芋仙, 杨枝甘露, 椰果, 元宵, 月饼, 芝麻糊, 芝士, 紫米糊, 粽	pancake, ice jelly, ice cream (*bingjilin*), ice cream (*bingjiling*), ice cream (*bingqilin*), ice cream (*bingqiling*), sorbet, biscuit, pudding, brownie, cake, egg tart, tofu pudding, bean paste, *guilinggao*, sugarcoated haws on stick walnut inside (*hetaojiaxin*), red dates white fungus (*hongzaoyiner*), peanut paste, caramel, silver lotus porridge (*yinerlianzi*), muffin (*mafen*), mousse, cheese (*nailao*), milkshake, cream, lotus root starch soup, chocolate, cookies, double skin milk, muffin (*songbing*), muffin (*songgao*), *tangyuan*, candied gourd, syrup, peach gum braised white fungus (*taojiaoyiner*), tiramisu, toast, *ximilao*, *ximilu*, grass jelly, *xianyuxian*, *yangzhiganlu*, coconut jelly, *yuanxiao*, mooncake, sesame paste, cheese (*zhishi*), purple rice paste, *zongzi*

© 2021 Agarwal S et al. *JAMA Network Open.*
